# Tyrosine kinase inhibitor SU6668 represses chondrosarcoma growth via antiangiogenesis *in vivo*

**DOI:** 10.1186/1471-2407-7-49

**Published:** 2007-03-17

**Authors:** Frank M Klenke, Amir Abdollahi, Elisabeth Bertl, Martha-Maria Gebhard, Volker Ewerbeck, Peter E Huber, Axel Sckell

**Affiliations:** 1 Department of Orthopedic Surgery, Inselspital, University of Bern, CH-3010 Bern, Switzerland; 2 Department of Radiation Oncology, German Cancer Research Center, INF 280, D69120 Heidelberg, Germany; 3 Division of Toxicology and Cancer Risk Factors, German Cancer Research Center, INF 280, D-69120 Heidelberg, Germany; 4 Department of Experimental Surgery, University of Heidelberg, INF 365, D-69120 Heidelberg, Germany; 5 Department of Orthopedic Surgery, University of Heidelberg, Schlierbacher Landstrasse 120a, D-69118 Heidelberg, Germany; 6 Department of Trauma and Reconstructive Surgery, Charité University Medical Center, Campus Benjamin Franklin, Hindenburgdamm 30, D-12200 Berlin, Germany

## Abstract

**Background:**

As chondrosarcomas are resistant to chemotherapy and ionizing radiation, therapeutic options are limited. Radical surgery often cannot be performed. Therefore, additional therapies such as antiangiogenesis represent a promising strategy for overcoming limitations in chondrosarcoma therapy. There is strong experimental evidence that SU6668, an inhibitor of the angiogenic tyrosine kinases Flk-1/KDR, PDGFRbeta and FGFR1 can induce growth inhibition of various primary tumors. However, the effectiveness of SU6668 on malignant primary bone tumors such as chondrosarcomas has been rarely investigated. Therefore, the aim of this study was to investigate the effects of SU6668 on chondrosarcoma growth, angiogenesis and microcirculation *in vivo*.

**Methods:**

In 10 male severe combined immunodeficient (SCID) mice, pieces of SW1353 chondrosarcomas were implanted into a cranial window preparation where the calvaria serves as the site for the orthotopic implantation of bone tumors. From day 7 after tumor implantation, five animals were treated with SU6668 (250 mg/kg body weight, s.c.) at intervals of 48 hours (SU6668), and five animals with the equivalent amount of the CMC-based vehicle (Control). Angiogenesis, microcirculation, and growth of SW 1353 tumors were analyzed by means of intravital microscopy.

**Results:**

SU6668 induced a growth arrest of chondrosarcomas within 7 days after the initiation of the treatment. Compared to Controls, SU6668 decreased functional vessel density and tumor size, respectively, by 37% and 53% on day 28 after tumor implantation. The time course of the experiments demonstrated that the impact on angiogenesis preceded the anti-tumor effect. Histological and immunohistochemical results confirmed the intravital microscopy findings.

**Conclusion:**

SU6668 is a potent inhibitor of chondrosarcoma tumor growth *in vivo*. This effect appears to be induced by the antiangiogenic effects of SU6668, which are mediated by the inhibition of the key angiogenic receptor tyrosine kinases Flk-1/KDR, PDGFRbeta and FGFR1. The experimental data obtained provide rationale to further develop the strategy of the use of the angiogenesis inhibitor SU6668 in the treatment of chondrosarcomas in addition to established therapies such as surgery.

## Background

Chondrosarcomas are the second most frequent malignant primary bone tumors in humans and are usually resistant to ionizing radiation and chemotherapy. To date, the only curative therapy is the surgical resection provided that an R0-situation can be achieved and metastases do not exist at the time of the diagnosis.

Due to the knowledge that a solid tumor cannot grow beyond a critical size of 1–2 mm^3 ^or metastasize without an adequate blood supply [1,2], therapeutic strategies targeting tumor vasculature, i.e. antiangiogenic therapies, represent a promising therapeutic option in addition to established therapies. Cells from the tumor vasculature are generally non-transformed and less prone to acquiring drug resistance [3]. Therefore, endothelial processes are considered as ideal targets for the prevention and control of tumor growth [4]. The idea of inhibiting tumor neovascularization without causing negative side-effects in the host vascular system is based further on the observation that the vasculature in normal adults is generally quiescent, with only 0.01% of endothelial cells undergoing cell division at any given time. Regarding tumor vasculature, the fraction of cycling endothelial cells may be 2–3 orders of magnitude higher. Thus, agents that antagonize active signal transduction are likely to have a minimal effect on the normal vasculature and therefore allow a more targeted approach regarding proliferating tumor vessels [5]. In principle, antiangiogenic therapy is applicable on any tumor entity, as endothelial cells represent a common cell type of all solid tumors [4]. Substances inhibiting active signal transduction of the angiogenic cascade, such as inhibitors of the receptor tyrosine kinases, have shown promising antiangiogenic effects in preclinical settings if administered as single agents or in combination with established therapies such as chemotherapy and radiation [6-12].

SU6668 is a small-molecule inhibitor of the receptor tyrosine kinases Flk-1/KDR (vascular endothelial growth factor receptor 2, VEGR2), PDGFRbeta (platelet-derived growth factor receptor beta) and FGFR1 (fibroblast growth factor receptor 1), which play an important role in angiogenesis, as they transduce the signals of the key angiogenic growth factors VEGF, PDGF and bFGF. Due to this mechanism, SU6668 is considered to be a potent substance for antagonizing central pathways of angiogenic signal transduction. The substance has shown promising antiangiogenic effects on primary tumors such as colon and lung carcinomas [9-12]. However, studies on the therapeutic efficiency of receptor tyrosine kinase inhibitors such as SU6668 on primary bone tumors are very rare [13]. The aim of the present study, therefore, was to investigate the *in vivo *effects of SU6668 on growth, angiogenesis and microcirculation of chondrosarcomas by means of intravital microscopy.

## Methods

### Animal model and cell lines

Experiments were performed on 10 adult male severe combined immuno deficient mice (SCID, C.B-17/IcrCrl-scid-BR, Charles River Laboratories Inc., Sulzfeld, Germany, 7 to 8 weeks old, 20 to 25 g body weight), following institutional guidelines approved by the local animal review board. All surgical procedures were performed in strictly aseptic conditions within a laminar flow unit (Merck Eurolab, Bruchsal, Germany) under deep anesthesia by an intra peritoneal injection of a mixture of ketamine (Ketanest^®^, 65 mg/kg body weight, Pfizer, Karlsruhe, Germany), xylazine (Rompun^®^, 13 mg/kg body weight, Bayer, Leverkusen, Germany) and acepromazine (Sedastress^®^, 2 mg/kg body weight, Medistar, Holzwickede, Germany).

The human chondrosarcoma cell line SW 1353 was obtained from ATCC (HTB-94, LGC Promochem GmbH, Wesel, Germany). Tumor cells [1 × 10^7^/ml] were injected subcutaneously into the left flank of each donor mouse and grown to a volume of 0.5 to 1.0 cm^3^. After sacrificing the donor mouse, the tumor was excised, cut into small chunks (volume 0.5–1.0 mm^3^) in Dulbecos Modified Eagle's Medium (DMEM,) at 4°C, and implanted into the recipient mouse in the following manner:

Surgical preparation of the cranial window was performed as described in detail elsewhere [14]. Briefly described, the scalp of the mouse was shaved and surgically excised in an oval area to expose the frontal and parietal bone. The periosteum was removed and an oval cavity of approximately 2 by 1 by 0.5 mm was milled into the calvaria by eliminating parts of the external tabula of the calvaria including the spongious bone underneath. Then one small chunk (approx. 0.5–1.0 mm^3^) of the human chondrosarcoma SW 1353 was implanted into the cavity. To prevent dehydration or mechanical damage to the tumors, the preparation was sealed with a glass cover slip and bone cement made of a mixture of ethyl cyanoacrylate glue (Pattex^® ^Blitz Kleber, Henkel, Germany) and GC Ostron^®^-Powder (methyl methacrylate polymer, GC Europe, Belgium).

The animals were housed individually in special filter cages to maintain aseptic conditions and to prevent mutual damage to the cranial window. The animals were provided with sterile standard pellet food and water *ad libitum*.

### SU6668 treatment

SU6668 was provided by Sugen Inc. (San Francisco, California, USA) and was dissolved in a carboxymethylcellulose (CMC)-based vehicle at 50 mg SU6668 per ml vehicle. Five animals each were treated by subcutaneous injection of 250 mg/kg body weight SU6668 (SU6668, n = 5) or the equivalent amount of the (CMC)-based vehicle alone (Control, n = 5) every 48 hours. The chosen SU6668 dosage is in accordance with the dosages for animal trials reported in the literature and represents the lower range of dosages that were shown to be effective against primary tumors [9,10,13,15,16]. Treatment started on day 7 after tumor implantation and continued until termination of the experiments on day 28 after tumor implantation.

### Intravital microscopy

For intravital microscopy, mice were anesthetized and positioned on a custom made stereotactic device.

In the first week after tumor implantation, mice were investigated daily under epi-illumination with a stereotactic microscope (Leica MZ7_5_, Leica, Germany) employing a 5 to 40 fold magnification. At 24-hour intervals, the first appearance of (i) hemorrhage, (ii) the first appearance of newly formed blood vessels entering the implanted tumor tissue, and (iii) the onset of perfusion in these newly formed vessels were determined.

Intravital fluorescence video microscopy was performed using an epi-illumination fluorescence microscope unit (Leica, Germany) equipped with a 4x (EF 4/0.12, Leitz, Wetzlar, Germany) and 40x (Zeiss Achroplan 40x/0.75 w, Carl Zeiss, Germany) objective on days 7, 14, 21, and 28 after tumor implantation. For *off line *analysis, regions of interest were recorded on video tapes using a S-VHS videocassette recorder (AG-7350, Panasonic, Japan) at a rate of 50 frames/s and a digital camera (Kappa CF 8/1, Kappa Opto-electronics, Germany).

Using an adequate fluorescence filter set for green light (bandpass 515–560 nm), the intravenous injection of the plasma marker fluorescein isothiocyanate (FITC)-labeled dextran (Sigma, St. Louis, MO, FITC-Dextran, FD 2000S, molecular weight 2.000.000; 0.1 ml of a 5% solution in 0.9% NaCl) enabled the observation of tumor microcirculation.

### Off-line analysis of tumor growth and microhemodynamics

Tumor growth was determined *off-line *by measuring its two-dimensional surface area in mm^2 ^from standardized digital photographs of the cranial window preparation at 10-fold magnification on days 7, 14, 21, and 28 after implantation using a computer-based analysis program (AnalySIS^® ^V3.0, Soft Imaging System, Münster, Germany).

The functional microvessel density (*FVD*) was determined as the length of all perfused microvessels within a tumor in relation to the two-dimensional surface area of the tumor in mm/mm^2 ^indicated by the fluorescence of FITC-labeled dextran in all perfused vessels. Recordings on video tape for *off-line *analysis of the FVD were made for 15s, each. *Off-line *analysis was performed using a computer-based image analysis program (CapImage^®^, Engineering Office Dr. Zeintl, Heidelberg, Germany).

### Histopathologic and immunohistochemical assessment

The mice were sacrificed on day 28 after tumor implantation and the tumors were immediately excised along with the surrounding tissue of the calvaria and the brain for further histopathologic and immunohistochemical investigations. Tissue samples were fixed for 24–48 hours by immersion in 4% formalin solution. After decalcification of the bone in ethylene-diaminetetraacetic acid for 2 weeks, samples were embedded in paraffin and sliced into three-μm serial sections for Hematoxylin-Eosin staining and five-μm serial sections for immunohistochemistry.

To identify endothelial cells, immunohistochemistry for CD31 (PECAM-1) was performed. After deparaffinization, sections were pretreated with Retrievagen A^® ^solution (Cat. No. 550524, BD Pharmingen, Basel, Switzerland) for antigen retrieval at 90°C for 10 minutes. Tissue sections were placed into 0.3% H_2_0_2 _in tris buffered saline (TBS) and 100% goat serum afterwards to block endogenous peroxidase and unspecific binding, respectively. Tissue samples were incubated overnight at 4°C with the primary antibody for CD31 (anti-mouse CD31 from rat, Cat. No. 550274, BD Pharmingen, Basel, Switzerland) or with mouse IgG for negative controls. Subsequently, the secondary antibody (biotinylated anti-rat IgG from goat, 1:1000, Cat. No. RPN1005, Amersham Biosciences, Otelfingen, Switzerland) and Streptavidin-horadish peroxidase conjugate (Cat. No. RPN1231, Amersham Biosciences, Otelfingen, Switzerland) were applied to the sections. Sections were then stained with 3-Amino-9-ethylcarbazole substrate (AEC, Cat. No. A6926, Sigma, Buchs, Switzerland), counterstained with hematoxylin and mounted with AquaTex.

### Statistics

All numerical data are presented as median with 25% and 75% quartiles. Using the software program SigmaStat^® ^for Windows (Version 2.03, SPSS, Chicago, IL), data were analyzed statistically. The Mann-Whitney rank sum test was applied for pair wise comparison procedures. Differences were considered significant at p < 0.05.

## Results

The first newly formed vessels in all tumors were observed 6 days after implantation. Vessel formation was followed by a rapid onset of perfusion within 24 hours. Intravital microscopy showed that the origin of angiogenic sprouting was from vessels located within the surrounding bone.

**Figure 1:**
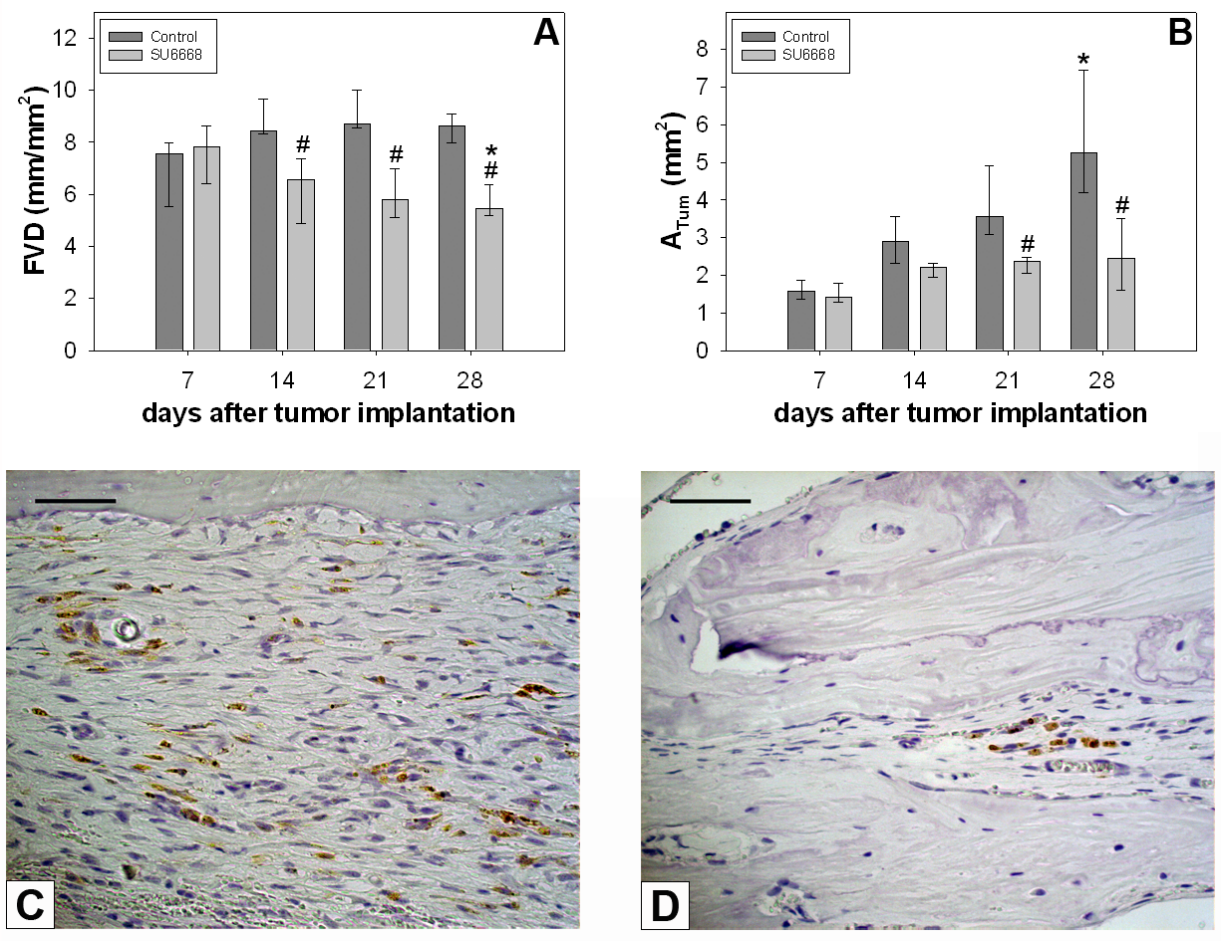
**A: **Functional vessel density, time course. Y-axis: functional vessel density (FVD in mm/mm^2^), x-axis: time in days after tumor implantation. SU6668 induced a regression of functional vessel density (FVD), while FVD in Controls increased between day 7 and 14 after tumor implantation, remaining constant thereafter. **B: **Tumor surface, time course. Y-axis: tumor surface (A_TUM _in mm^2^), x-axis: time in days after tumor implantation. In Controls, tumor surface continuously increased over the period of investigation. In contrast to this, SU6668 induced a growth arrest of chondrosarcomas beginning on day 14 after tumor implantation. **A and B: **Treatment started on day 7 after tumor implantation. Results are given as median with 25% quartiles and 75 % quartiles. Mann-Whitney rank sum test, ^#^p < 0.05 versus Control, *p < 0.05 versus day 7 after tumor implantation. **C and D: **Immunohistochemistry for CD31 (PECAM-1) on day 28 after tumor implantation confirmed the results of the *in vivo *quantification of the functional vessel density with intravital microscopy. Untreated Controls showed uniformly distributed CD31 positive cells (brown) within the implanted tumors (**C**). In SU6668 treated tumors, CD31 positive cells were rare and only detectable in peripheral areas of the tumor residues (D). Scale bars represent 50 μm.

In Controls, functional vessel density (FVD) increased between day 7 and day 14 after tumor implantation, reaching a constant plateau between days 14 and 28 (Fig. 1a). After the initiation of therapy on day 7 after tumor implantation, FVD continuously decreased over the period of investigation with SU6668 (day 28: 5.5 mm/mm^2 ^(5.1/6.3) vs. day 7: 7.8 mm/mm^2 ^(6.7/8.3); Fig. 1a). On day 28 after tumor implantation, FVD was significantly smaller with SU6668 compared to Controls (5.5 mm/mm^2 ^(5.1/6.3) vs. 8.6 mm/mm^2 ^(8.1/8.9); Fig. 1a).

Tumor growth was identical in all animals until day 7 after tumor implantation. In Controls, the two-dimensional tumor surface (A_Tum_) continuously increased over the period of investigation reaching 5.3 mm^2 ^(4.4/7.2) on day 28 after tumor implantation (Fig. 1b). After initiation of the treatment on day 7 after tumor implantation, in SU6668 A_Tum _increased until day 14 after tumor implantation. Subsequently to day 14 after tumor implantation, a growth arrest of chondrosarcomas occurred in SU6668. On day 28 after tumor implantation, the A_Tum _was 2.5 mm^2 ^(1.9/3.4) in SU6668, thus being statistically smaller than in Controls, where A_Tum _was 5.3 mm^2 ^(4.4/7.2; Fig. 1b).

The comparison of the time courses of FVD and A_Tum _demonstrated that in SU6668, A_Tum _had still increased in the first week after therapy initiation, while FVD was already regressive over the same time period of investigation.

**Figure 2:**
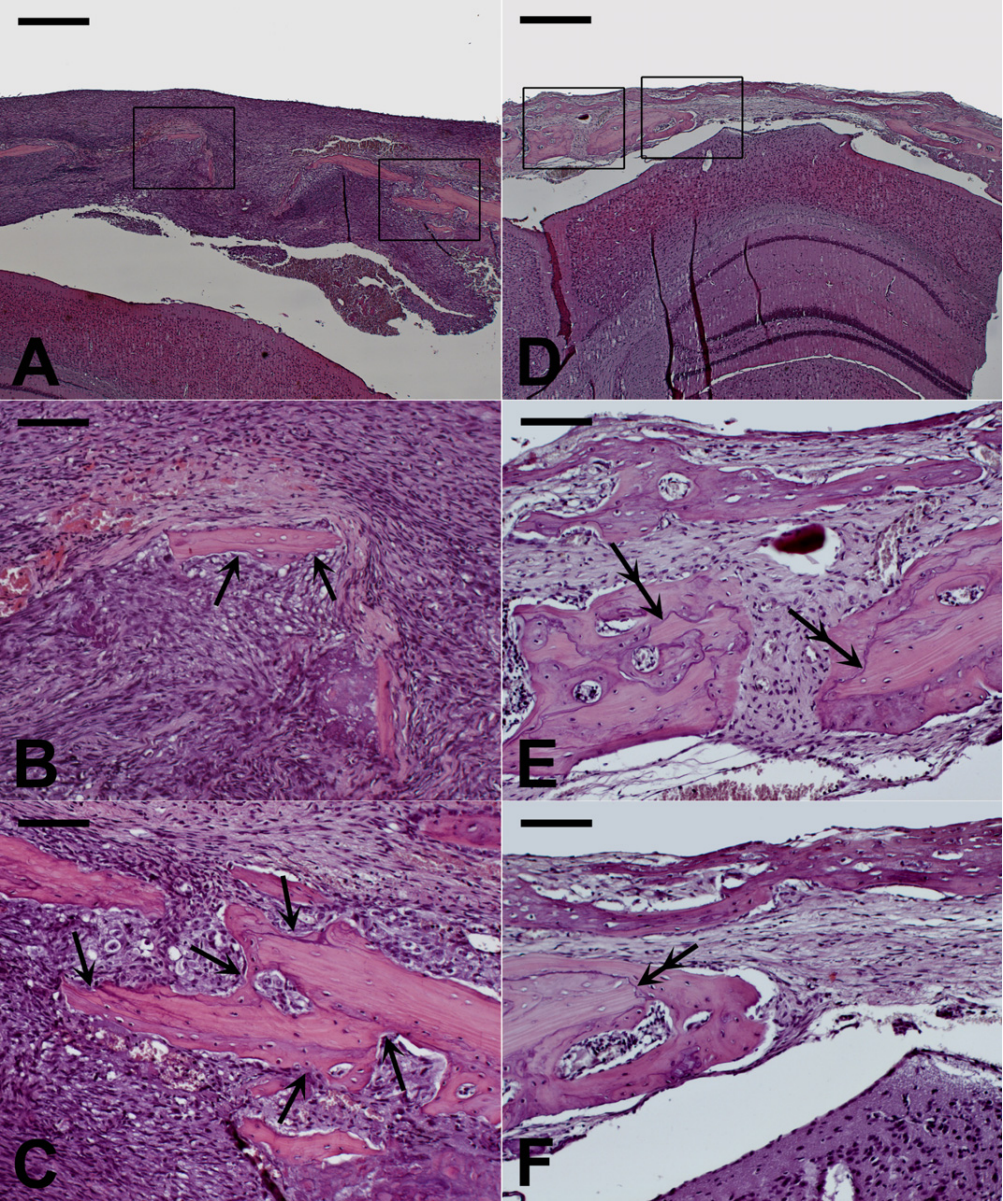
SW 1353 chondrosarcoma at day 28 after tumor implantation, hematoxylin-eosin stained cross section from representative tissue samples of the cranial window preparation. **A, D: **4-fold magnification, scale bar 500 μm. **B, C, E, F: **20-fold magnification, scale bar 100 μm. In Controls, the overview magnification shows a large tumor formation with high cellular density (**A**). Enlarged cutaway views (**B, C**) from the framed regions show high cellular density and destructive tumor growth with resorption of the surrounding bone (arrows, tips pointing to resorption lacunae). In SU6668, tumor size was markedly reduced (**D**). Enlarged cutaway views (**E, F**) from the framed regions show fibrous "scar" tissue with residual tumor cells and low cellular density and regenerative bone formation at sites of previous bone resorption (double arrows, distal tip pointing to former resorption front, proximal tip pointing to new mineralization front).

Histologically, typical signs of a malignant tumor were observed in Controls on day 28 after tumor implantation. In accordance to *in vivo *findings, the volume of chondrosarcomas increased significantly compared to the small chunks (volume 0.5–1.0 mm^3^) that were initially implanted (Fig. 2a). Large tumor masses had grown above, below, and into the calvaria. Histology revealed extensive infiltration and resorption of the adjoining bone as signs for typical growth behavior of malignant tumors. However, infiltration into the underlying brain was not observed on day 28 after tumor implantation (Fig. 2a–c). In contrast to this, the volume of chondrosarcomas in SU6668 remained at a much smaller level compared to Controls. Furthermore, tumors showed signs of regression in SU6668. On day 28 after tumor implantation, there was not one large formation of any solid tumor, but residual chondrosarcoma cells were observed embedded in fibrous tissue that filled large parts of the cranial window preparation (Fig. 2d–f). In regions of previous bone resorption, regenerative bone formation was observed (Fig. 2e). The immunohistochemical staining of endothelial cells confirmed the results of the *in vivo *quantification of the functional vessel density with intravital microscopy. Untreated tumors showed uniformly distributed CD31 positive endothelial cells. In contrast, tumors which were treated with SU6668 were only weekly positive for CD31 (Fig. 1c–d).

Injections of SU6668 or of the CMC-vehicle alone were well tolerated, as no differences in animal behavior or loss of weight were observed.

## Discussion

Due to the insufficient response of chondrosarcomas to chemotherapy and ionizing radiation, current curative therapy of these tumors is usually limited to surgery. As angiogenesis is essential for tumor growth, the selective destruction of tumor feeding and draining blood vessels represents a promising strategy for overcoming the therapeutic limitations of malignant primary bone tumors.

The small molecule receptor tyrosine kinase inhibitor SU6668 was used as an antiangiogenic agent against the human chondrosarcoma SW1353, which was grown in a cranial window preparation in SCID mice. SU6668 mediates the simultaneous inhibition of the receptor-tyrosine kinase activity of vascular endothelial growth factor (VEGF), fibroblast growth factor (FGF), and platelet-derived growth factor (PDGF), which are key mediators of tumor angiogenesis [9,15,17-19]. SU6668 has already shown encouraging antiangiogenic effects on some solid tumors [9-11]. However, the effects of SU6668 on primary bone tumors have only been described once for Ewing's sarcoma family tumors [13].

The present data demonstrate the effective anti-tumor action of SU6668. Treatment of SW1353 chondrosarcomas with SU6668 at 250 mg/kg/48 h decreased the tumor size to 53% of the size in Controls on day 28 after tumor implantation. The time course of two-dimensional tumor surfaces demonstrated that only SU6668 induced a growth arrest of tumors starting on day 14 after tumor implantation. In Controls, at the same time a continuous increase of the tumor size was measured. The *in vivo *findings were confirmed by histology from tissues excised on day 28 after tumor implantation. In Controls, typical signs of the behavior of malignant tumor growth were observed while SU6668 treatment resulted in small residual tumors with the formation of scar tissue within the tumor. These results are consistent with previous findings on growth inhibition of tumor xenografts by SU6668: Laird et al. [15] found a slight increase in volumes of the human epidermoid tumor xenograft A431 within the first week after initiation of SU6668 treatment resulting in a growth arrest of these tumors thereafter. Marzola et al. [10] described a 60% growth inhibition of HT-29 colon carcinomas treated with SU6668 compared to untreated Controls.

The anti-tumor activity of SU6668 has been attributed to its antiangiogenic potential arising from its inhibitory effect on the receptor-tyrosine kinase activity of VEGF, FGF, and PDGF. Inhibition of the downstream action of these angiogenic receptors has been shown to induce endothelial cell apoptosis [9,20]. Furthermore, Laird et al. [9] showed that endothelial cell apoptosis preceded tumor cell apoptosis. The authors concluded that SU6668 treatment initiates vessel death by apoptosis, which in turn leads to subsequent tumor cell death. In the present study, SU6668 significantly reduced functional vessel density of tumors by 37% compared to Controls, demonstrating the antiangiogenic potential of SU6668. A comparison of the time courses of functional vessel density and tumor size demonstrated that SU6668 primarily induced a reduction in tumor vascularization directly after initiation of this therapy. This was followed by growth arrest of the implanted chondrosarcomas starting on day 14 after tumor implantation. In accordance with findings by Laird et al. [9], and analogous to findings from a previous study where Celecoxib-induced inhibition of the functional vessel density caused a reduced growth of the lung carcinoma A 549 implanted into a cranial window preparation [21], we conclude that an underlying mechanism of the anti-tumor effect of SU6668 is the antiangiogenic potential resulting in a reduction in blood vessels that feed and drain tumors, which in turn halts tumor progression.

Although the tumor vasculature is thought to be little prone to acquiring drug resistance [3,22,23] recurrence of tumor growth after an initial period of suppression during long-term antiangiogenic therapy has also been described [24-26]. Acquired drug resistance has predominantly been described for drugs only interfering with a single angiogenic pathway [24-26]. As SU6668 does not target just one signaling pathway but inhibits signal transduction through the growth factor receptors VEGFR2, PDGFb2 and FGFR1, drug resistance is less likely to occur [24].

## Conclusion

The data presented demonstrate that SU6668 is a potent inhibitor of tumor growth of chondrosarcomas *in vivo*. The anti-tumor effects can be attributed to the antiangiogenic effects of the substance, which are mediated by the inhibition of the receptor tyrosine kinases Flk-1/KDR (VEGR2), PDGFRbeta and FGFR1. Antiangiogenic therapy using the application of SU6668 appears to be a promising way to expand the therapeutic options in chondrosarcoma therapy. However, long-term studies must be performed to elucidate the effects of a long-term treatment such as potential side effects, the development of drug resistance or relapse of tumor growth.

## Competing interests

The author(s) declare that they have no competing interests.

## Authors' contributions

FMK participated in designing the study, carried out the animal experiments, the histology and immunohistochemistry, interpreted the data, and drafted the manuscript. MMG and VE participated in designing the study and revising the manuscript critically. AA and PEH established the SW 1353 cell cultures and tumor cell suspensions and participated in revising critically the manuscript. EB carried out data acquisition from the *in vivo *experiments. AS conceived, coordinated, and designed the study, performed the statistical analysis, interpreted the data, and drafted the manuscript. All of the authors have read and approved the final manuscript.

## Pre-publication history

The pre-publication history for this paper can be accessed here:
